# Arabidopsis AGAMOUS Regulates Sepal Senescence by Driving Jasmonate Production

**DOI:** 10.3389/fpls.2017.02101

**Published:** 2017-12-11

**Authors:** Rubina Jibran, Jibran Tahir, Janine Cooney, Donald A. Hunter, Paul P. Dijkwel

**Affiliations:** ^1^The New Zealand Institute for Plant and Food Research Limited, Palmerston North, New Zealand; ^2^Institute of Fundamental Sciences, Massey University, Palmerston North, New Zealand; ^3^The New Zealand Institute for Plant and Food Research Limited, Hamilton, New Zealand

**Keywords:** abscission, AGAMOUS, *ag-1*, *delayed dehiscence 2* (*dde2*), *defective in anther dehiscence 1* (*dad1*), flower, jasmonate, senescence

## Abstract

The signal that initiates the age-regulated senescence program in flowers is still unknown. Here we propose for the ephemeral *Arabidopsis thaliana* flower that it dies because of continued expression of the MADS-box transcription factor AGAMOUS (AG). AG is necessary for specifying the reproductive structures of the flower. Flowers of *ag-1*, which lack AG, exhibited delayed sepal senescence and abscission. The flowers also had reduced jasmonic acid (JA) content. Other anther-defective sterile mutants deficient in JA, *defective in anther dehiscence 1* (*dad1*) and *delayed dehiscence 2* (*dde2*), exhibited delayed sepal senescence and abscission as well. Manually pollinated *dad1* flowers produced siliques but still had delayed senescence, demonstrating that absence of pollination does not cause delayed senescence. When *ag-1, dad1* and *dde2* flowers were sprayed with 100 μM methyl jasmonate, the sepal senescence and abscission phenotypes were rescued, suggesting that JA has a role in these processes. Our study uncovers a novel role for AG in determining the timing of death of the flower it helps develop and highlights a role for JA in sepal senescence.

## Introduction

Flower longevity varies widely among species. Some flowers last only a few hours before dying, whereas others may persist for several months before they senesce (Stead, 1992). It is still unknown how senescence is initiated in flowers (van Doorn and Woltering, 2008). In some flowers ethylene is important (Woltering and van Doorn, 1988), with the hormone being produced in the gynoecium following pollination (Hunter et al., 2004; van Doorn and Woltering, 2008). Other flowers senesce independently of pollination and an associated ethylene burst (van Doorn, 1997). For ephemeral flowers (flowers lasting less than a day), no clear effect of pollination on longevity has been found (van Doorn, 1997).

MADS-box proteins are transcription factors, which have been integral to the evolution of complexity and reproductive fitness in plants (Smaczniak et al., 2012). Phylogenetic analyses of MADS-box genes in green algae suggested that they first appeared ∼500 million years ago in branched filamentous charophycean algae, from which land plants evolved (Tanabe et al., 2005). They bind to promoter DNA sequences, having high similarity with the motif CC(A/T)_6_GG known as the CArG-box (Theissen, 2001). MADS-box proteins regulate many plant-developmental processes including flower senescence, floral organ specification, gametophyte, embryo and seed development (Smaczniak et al., 2012; Chen et al., 2015). They also influence two age-related maturity transitions in plant tissues that are essential for the plant to complete its life cycle successfully: the juvenile-to-adult (vegetative phase) and adult-to-reproductive (flowering phase) transitions (Deng et al., 2011; Huijser and Schmid, 2011).

The MADS-box family of proteins are perhaps best known for their role in specifying floral identity in the ABCDE model of flower development. In this model floral homeotic genes are classified into five functional classes (A-E). Class A genes are *APETALA1* and *APETALA2*, B are *APETALA3* and *PISTILLATA*, C is *AGAMOUS*, D are *FLORAL-BINDING PROTEIN 7* and *11*, and E are *SEPALLATA1-4* (Pelaz et al., 2000; Honma and Goto, 2001; Smaczniak et al., 2012). The identities of the four whorls of the flower are governed by the overlapping expression of the above-mentioned MADS-box genes. Mutations in these genes result in floral homeotic abnormalities (Irish and Sussex, 1990). For example, sepals are transformed into leaf-like organs when A class activity of *APETALA1* and *APETALA2* is lost.

Results from ectopic expression studies of MADS box genes suggest that this family of transcription factors can also control the timing of flower senescence. For example, ectopically expressed AGAMOUS-LIKE 15 (AGL15) and AGL18 delay sepal senescence and perianth abscission in *Arabidopsis thaliana* (Arabidopsis) (Fernandez et al., 2000; Adamczyk et al., 2007). Similarly, when *AGL42* was constitutively expressed in Arabidopsis, floral senescence was delayed through suppression of components in the ethylene signaling pathway (Chen et al., 2011, 2015). Thus, MADS-box proteins are not only involved in specifying the complex architecture of plants, but may also be involved in controlling their eventual fate.

The MADS-box gene *AG* was identified by Yanofsky et al. (1990) after a T-DNA insertion mutant was found to display a similar phenotype to the ethyl methanesulfonate mutant *ag-1*, first described by Koornneef et al. (1983). In addition to its well-described role in specifying stamen organ identity in floral primordia, it is now known that AG continues to be expressed in stamens and directly activate the jasmonic acid (JA) biosynthesis gene, *DEFECTIVE IN ANTHER DEHISCENCE 1* (*DAD1)* (Ito et al., 2007). Ishiguro et al. (2001) suggested that JA drives anther development and floral petal and sepal expansion by regulating water transport in stamens and petals.

Senescence functions to ensure efficient removal of nutrients from dying parts of the plant to the flowers, fruit and grains (Gan and Amasino, 1997). Better understanding of the key regulators of senescence will help to maximize crop yield and improve quality of fruits, vegetables and cut flowers. The role of endogenous JA in controlling age-related senescence is not clear (Jibran et al., 2013). For although senescence of detached leaves in many plant species is accelerated by exogenously applied methyl jasmonate (MeJA) or JA, leaf senescence is not delayed in JA biosynthesis mutants such as *allene oxide synthase* (*aos*)/*delayed dehiscence 2* (*dde2*) (Park et al., 2002) and *oxophytodienoate reductase 3* (*opr3*) (Schommer et al., 2008). Perianth abscission does, however, appear to be regulated by JA in Arabidopsis (Kim et al., 2013).

Here we propose an additional role for AGAMOUS in flower development, suggesting that it is key for controlling lifespan of the flower. In our model, AGAMOUS specifies stamen and carpel identity and later controls senescence of the flower by directly activating *DAD1* expression in maturing stamens to produce JA.

## Materials and Methods

### General Plant Growth Conditions

Seeds were stratified at 4°C in the dark for 3–4 days. Mutants and wild type seeds of L*er*-0 and Col-0 were sown in wet seed Raising Mix^TM^ (Oderings, New Zealand) in a temperature-controlled growth chamber (20–22°C, 16-h/8-h light/dark cycle, white light ∼180 μE and 60% relative humidity, unless otherwise stated). The T-DNA insertion mutants *dad1* (SALK_025432.28.25.X), transposon insertion *dde2* (CS65993), and EMS mutant *ag-1* (CS19985) were purchased from The Arabidopsis Information Resource^1^.

### Phenotypic Analysis

Sepal senescence and abscission phenotypes of the mutant plants were compared with their respective wild types. To study senescence and abscission events, flower positions were defined according to the numbering system of Bleecker and Patterson (1997), which identifies flower position 1 as the top-most flower with visible white petals. In addition, sepal senescence and abscission was described using the developmental stages of flowers according to Alvarez-Buylla et al. (2010).

To quantify the onset of sepal senescence for Figures (**Figures 4B**, **5B** and Supplementary Figures S1–S5) the flowers of a single inflorescence were placed on a black background and photographed. The photograph was subsequently analyzed using Image J software. In each photograph, all buds/flowers were placed linearly in order from youngest to oldest. Five contiguous flowers were identified in this ordered set defined by the sepals of the first flower (Flo1) being completely green and the sepals of the last flower (Flo5) being completely yellow. The onset of senescence in the five flowers was then determined by the following method, for those flowers exhibiting yellowing during the time period the experiment was performed.

The color intensity of the sepals from each of the five candidate florets was quantified by recording mode values in Image J. The process for obtaining mode values for each sepal was as follows: (1) The photograph containing the five flowers was imported into Image J and converted into a red spectrum channel/ multi-channel composite image. (2) The sepal area of each flower was selected using polygon selection. (3) The color intensity of this selected area was analyzed with the histogram function. The peak of the histogram represented the intensity of the color present in the area. The mode value of the histogram peak was used as a quantitative measure of the color for sepals of each flower.

Once the mode values were acquired, the colour difference ratio (CDR) of the sepals of adjacent flowers was measured, i.e., this was a measure of the change in color between the developmental stages of adjacent flowers. For example, CDR1 = Mode value of Flo1 sepal/Mode value of Flo2 sepal. For a set of five flowers, four different CDR values were generated from which the maximum CDR value was considered to be the point of onset of sepal senescence, i.e., the maximum change in color from green to yellow.

### Jasmonic Acid Treatment

Six and eight-week-old plants were grown in long-day conditions (16-h light and 8-h dark) in the plant house and sprayed with 100 μM MeJA (Sigma–Aldrich NZ), dissolved in 1% (v/v) ethanol, each day for 3 days. Sepal senescence and abscission events were visually noted for the flowers from primary inflorescences of developmental stage 13, when the petals become visible between the sepals and continue to elongate rapidly (Alvarez-Buylla et al., 2010). This stage is before flower developmental stage 14 when the flower opens after pollination. For determining the effect of MeJA treatment on sepal senescence and abscission, flowers of 6-week-old plants at stage 13 were tagged with a cotton thread at day 0 of treatment and these tagged flowers were designated as ‘T’. The change in color from green to yellow of the T flowers and the flowers adjacent to them was analyzed for sepal senescence and abscission phenotypes 3 days following the MeJA or mock treatments [1% (v/v) ethanol]. The adjacent flowers were designated either positive or negative depending on their position relative to that of the tagged flower ‘T’, i.e., flower T^-1^ was the younger flower adjacent to flower T and was located above the T flower on a stalk, while flower T^+1^ was the older flower adjacent to flower T and was located below the T flower.

### Ethylene Measurement

Ethylene was quantified with a laser-based ETD-300 ethylene detector (Sensor Sense BV, Nijmegen, The Netherlands). The stop and flow method was used to measure ethylene. In this method, selected cuvettes (2 mL screw-capped closed tubes) were first flushed with air for 30 min and then the detached primary inflorescences (∼100 mg), from 8-week-old L*er*-0 and *ag-1* plants, placed in the tubes. Two needles (serving as inlet and outlet) were inserted into the top of the microfuge tube and instrument tubing fitted to the needles. Air that had passed through a catalyser to remove hydrocarbons entered the cuvette (through the inlet) and then carried the ethylene produced by the tissue to a scrubber (to remove water and CO_2_) and then onto the ethylene detector.

### Hormone Measurements

A 1000 μg mL^-1^ stock solution of JA, abscisic acid (ABA), salicylic acid (SA), and 1-aminocyclopropane-1-carboxylic acid (ACC) was prepared in methanol. A 10 μg mL^-1^ labeled internal standard stock solution of the isotopically labeled analogs, [^2^H_5_]JA, [^2^H_4_]SA and [^2^H_6_]ABA, was also prepared in methanol. A set of 0.1, 1, 10, 100, and 1000 ng mL^-1^ calibration standards of JA, SA, ABA, and ACC were made by diluting each stock solution with 1:1 methanol:water. The labeled internal standard solution mix (25 ng) was aliquoted into 400 μL of each calibration standard. Twenty five ng of the labeled internal standard solution mix and 400 μL of the extraction solvent (20:79:1 methanol:isopropanol:acetic acid) was added to 110 mg FW of freeze-dried tissue kept on ice. Each of the samples were then shaken for 30 min at 4°C and stored overnight -20°C. Next morning the samples were centrifuged at 13,000 × *g* for 10 min at 4°C and then 100 μL of the supernatant diluted 10-fold with water and analyzed by Liquid Chromatography Mass Spectrometry (LC-MS).

LC-MS analysis was performed using a 5500 QTrap triple quadrupole/linear ion trap (QqLIT) mass spectrometer fitted with a TurboIon-Spray^TM^ interface (AB Sciex, Concord, ON, Canada) and Ultimate 3000 UHPLC (Dionex, Sunnyvale, CA, United States). A kinetex column (Phenomenex, Torrance, CA, United States) at 60°C was used for the chromatographic separation. Water +1% formic acid (A) and acetonitrile +0.1% formic acid (B) were used as solvents at flow rate of 600 μL min^-1^. To identify JA, SA, and ABA, the initial mobile phase 15% B was ramped linearly to 50% B at 8 min, then ramped to 100% B at 8.1 min before being held for 2 min and resetting to the original conditions. An injection volume of 50 μL was used. Data was collected in the negative mode by using a multiple reaction monitoring (MRM) method. To analyze ACC, the initial mobile phase 10% B was ramped linearly to 50% B at 10 min before being held for 2 min and resetting to the original conditions. Data was collected in the positive mode using a multiple reaction monitoring (MRM) method. The Q1 and Q3 transitions monitored together with their optimized parameters (declustering potential (DP), entrance potential (EP), collision energy (CE) and collision cell exit potential (CXP) are listed in the Supplementary Table S1.

Additional operating parameters were: dwell time of 60 ms, ionspray voltage of -4500 V for negative mode and 4500 V for positive mode, temperature at 600°C, curtain gas of 45 psi, ion source gas 1 of 60 psi, ion source gas 2 of 60 psi. JA, SA, and ABA was quantified by the internal standard ratio method and ACC by external calibration.

### Chlorophyll Measurement

The chlorophyll concentration of detached uncrushed inflorescences and sepals was measured by holding the tissue in 96% (v/v) ethanol (10 mg of tissue per 30 μL of ethanol) for 4 days in the dark at 4°C. An aliquot of 2 μL of the supernatant was measured with a Nanodrop ND-1000 spectrophotometer using absorbances at wavelengths 649 and 665 nm and calculations were performed according to Wintermans and De Mots (1965).

$$Chlorophyll\,a=13.70\,A665nm-5.76\,A649nm\,\mu g\,{mL}^{-1}$$

$$Chlorophyll\,b=25.80\,A649nm-5.76\,A665nm\,\mu g\,{mL}^{-1}$$

$$Total\,Chlorophyll=6.1\,A665nm+20.04\,A649nm\,\mu g\,{mL}^{-1}$$

The chlorophyll content of each line or treatment from day 0 to the other days was compared with that of wild type. Student’s *t*-test was applied for statistical analysis of the regression parameter estimates.

## Results

### *ag-1* Flowers Show Delayed Sepal Yellowing and Abscission

**FIGURE 1:**
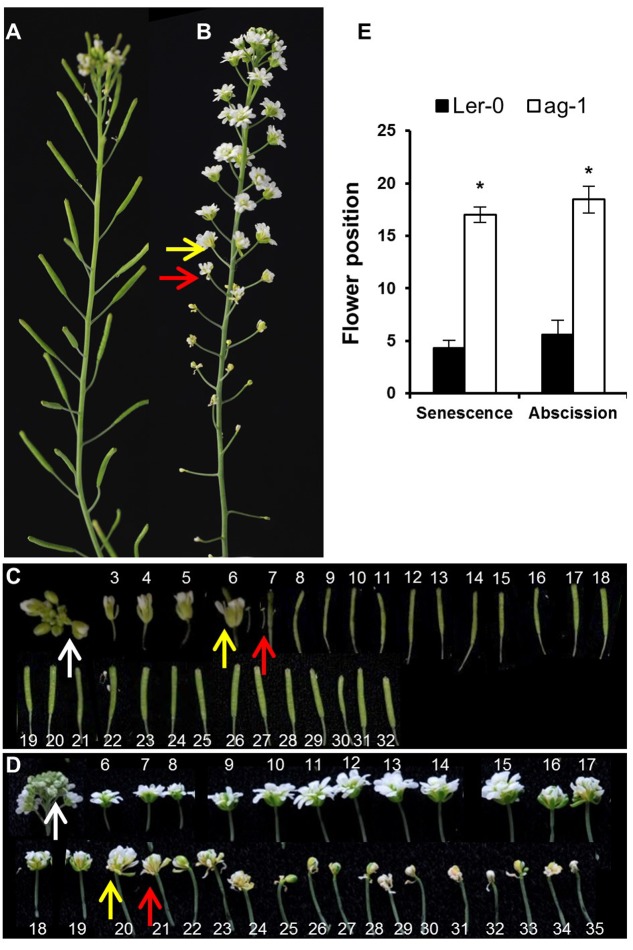
Yellowing and abscission of sepals in L*er*-0 and *ag-1* plants. **(A)** 8 week-old L*er*-0 plant. **(B)** 8 week-old *ag-1* plant. **(C)** L*er*-0 flowers detached from 8 week-old plant. **(D)** *ag-1* flowers detached from 8 week-old plant. **(E)** Position of flowers showing yellowing and abscission in 8 week-old L*er*-0 and *ag-1* plants. Plants were grown under long-day conditions for 8 weeks in a growth room (20–22°C, 16-h light and 8-h dark cycle, white light ∼180 μE and 60% relative humidity) and flowers were detached and subsequently photographed. The yellow and red arrows indicate flowers with senesced and abscised sepals, respectively. The white arrows indicate the flowers used for quantifying delayed sepal senescence for **Figure 2D**. The flowers are numbered according to the numbering system of Bleecker and Patterson (1997). The numbers in **(C,D)** indicate the positions of the flowers/siliques along the flowering stalk: flower 1 is the first opened flower present at the top, whereas the high numbers are the flowers/siliques present at the bottom of the stalk. The *ag-1* flower is composed of a repeating series of sepal-petal-petal and when whorls of petals abscises, the inner sepal whorl, green in color, appears. This can be seen by reduction in the *ag-1* flower size after flower position 20 in **(D)**. The photographs shown are representative of six to eight biological replicates. The error bars indicate standard errors of the mean of eight biological replicates. ^∗^Indicates statistically significant (*p* < 0.05) between L*er*-0 and *ag-1* using Student’s *t*-test.

Flower opening of wild type L*er*-0 Arabidopsis plants was first initiated after ∼ 5 weeks of growth that is almost similar to the time of first flower opening in Col-0 as described by Boyes et al. (2001). After 6 weeks, wild type L*er*-0 plants displayed many siliques with senesced and abscised floral organs, whereas none of the *ag-1* flowers (defective in AGAMOUS) showed senescence or floral organ abscission (Supplementary Figures S1, S2). Furthermore, it was clear that opening of *ag-1* flowers was delayed with petals not clearly expanding out of bud until their pedicels were well clear of the compressed inflorescence (Supplementary Figure S1). This was consistent with what has been previously reported by Ishiguro et al. (2001). However, by 8 weeks, the flowers of the *ag-1* inflorescences did not appear to be delayed in opening as the plants had many opened flowers within the compressed inflorescence (**Figure 1B**).

Arabidopsis flowers of 8-week-old *ag-1* plants (grown in long-day conditions) exhibited a dramatic delay in sepal yellowing and abscission compared with the wild type L*er*-0 (**Figure 1**). Sepal yellowing occurred at flower positions 3 to 7 in the wild type (**Figures 1A,C,E**) and position 11 to 24 in *ag-1* (**Figures 1B,D,E**) according to the numbering system of Bleecker and Patterson (1997), which defines flower position 1 as the top-most flower with visible white petals. The *ag-1* sepals did not abscise until flower positions 12–22 compared with flower positions 4 to 8 of the wild type (**Figures 1A,C,D**). Thus, lack of AG activity caused delayed sepal yellowing and abscission.

**FIGURE 2:**
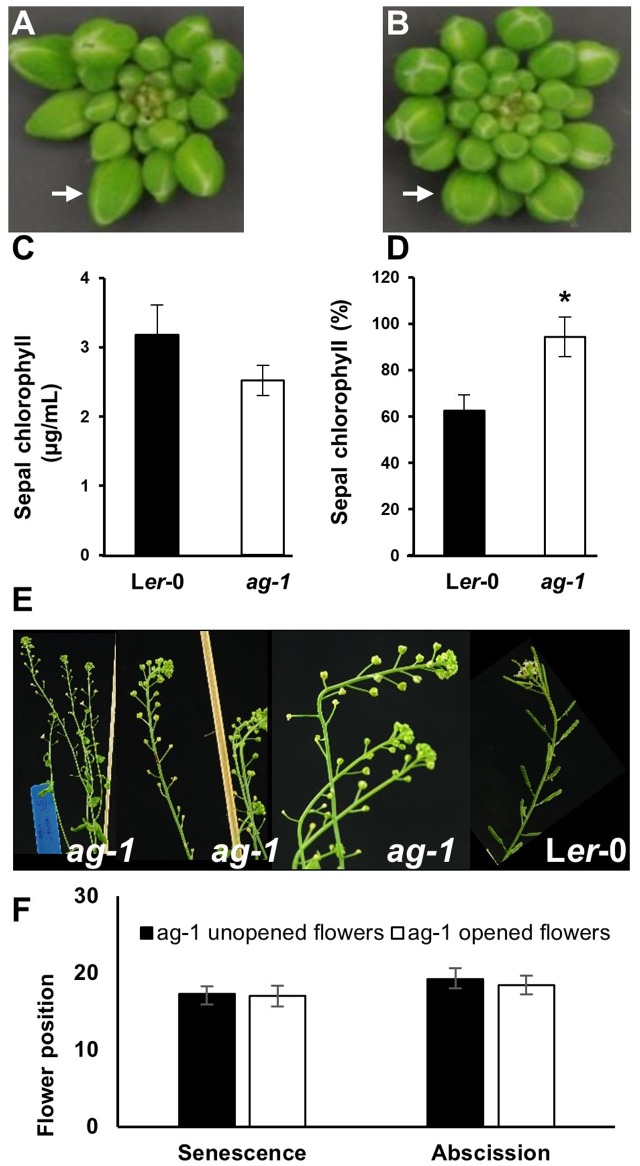
Sepal Yellowing of *ag-1* and L*er*-0 flowers. **(A)** L*er*-0 inflorescence. **(B)** a*g-1* inflorescence. **(C)** Chlorophyll concentration of L*er*-0 and *ag-1* sepals obtained from outermost buds. **(D)** Percentage chlorophyll of L*er*-0 and *ag-1* sepals at flower position 2 (indicated by white arrows in **Figures 1C,D**) compared with that in sepals from unopened flowers of the outer whorl shown here in **(A,B)**, indicated by white arrows. Sepal chlorophyll percentage was calculated by comparing the chlorophyll concentration of sepals from flowers at position 2 to that in sepals from unopened flowers. Plants were grown under long-day conditions for 8 weeks as described in Section Materials and methods. **(E)** 8-week-old *ag-1* plants with un-opened flowers. **(F)** Position of flowers showing yellowing and abscission in 8 week-old-*ag-1* plants with opened and un-opened flowers. 8-week-old *ag-1* plants with un-opened flowers were grown in a growth chamber with16-h-light/8-h-dark cycle, 100 μmol m^-2^ s^-1^ white light, and 60% relative humidity. The flowers are numbered according to the numbering system of Bleecker and Patterson (1997). The error bars indicate standard error of the means of six biological replicates. ^∗^Indicates statistically significant (*p < 0.05*) between the wild type (L*er*-0) and *ag-1* using Student’s *t*-test.

To quantify the difference in yellowing between *ag-1* and wild type and determine if the differences can be seen at the early stages of flower development, chlorophyll concentration was measured in the sepals taken from wild type and *ag-1* newly opened flowers (flower position 2, indicated by the white arrows in **Figures 1C,D**) and comparing it to the chlorophyll concentration of sepals from the outermost buds of unopened inflorescences (indicated by white arrows in **Figures 2A,B**). Sepal chlorophyll concentration of the *ag-1* unopened outermost bud was not significantly different from that of wild type (**Figure 2C**). However, *ag-1* sepals from opened flowers showed a reduced loss in chlorophyll (∼6%) compared with the wild type (∼40%) (**Figure 2D**). This suggested that lack of AGAMOUS activity in *ag-1* affected chlorophyll degradation but not accumulation or synthesis.

When *ag-1* plants were grown in a different environment at a different time, the flowers of older plants failed to open completely (**Figures 2E,F**). The reason for this is unclear, but despite this, the sepals of unopened *ag-1* flowers yellowed and abscised at similar flower positions (**Figures 2E,F**) as observed for sepals of *ag-1* flowers that opened (**Figures 1B,D,E**). Thus flower opening is not a prerequisite for sepal yellowing and abscission of *ag-1* flowers; rather flower opening and sepal senescence and abscission appeared to be independently regulated.

### *ag-1* Inflorescences Have Less JA than the Wild Type

**FIGURE 3:**
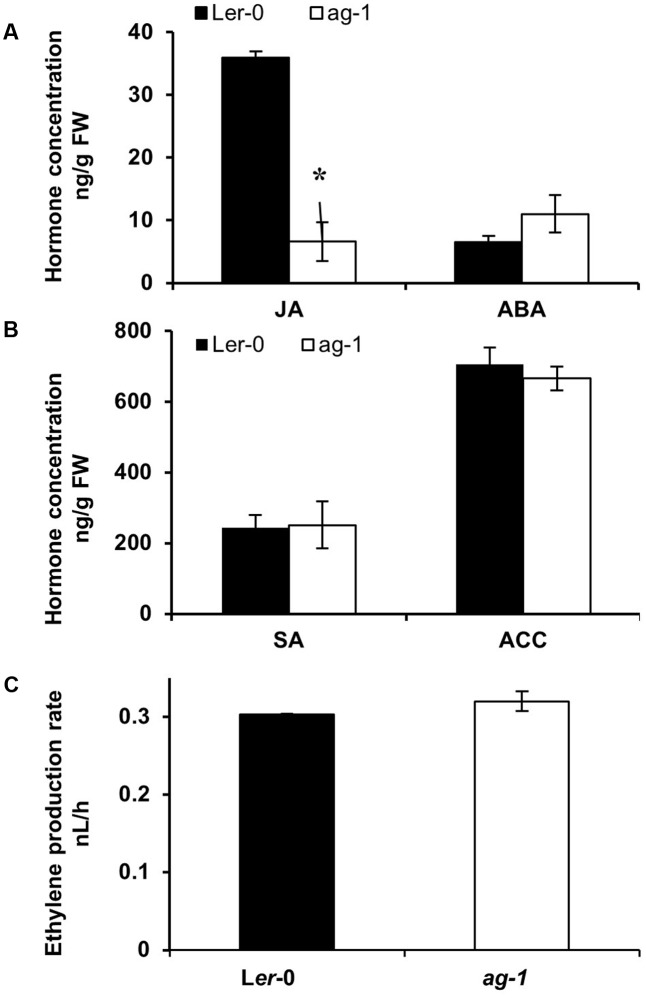
Endogenous hormone concentrations in L*er*-0 and *ag-1* inflorescences. **(A,B)** Endogenous hormone concentrations in L*er*-0 and *ag-1* inflorescences. **(C)** Ethylene evolution in detached inflorescences. Plants were grown under long-day conditions for 8 weeks and flowers were detached and sepals were snap frozen for hormone analyses. Jasmonic acid (JA), 1-Aminocyclopropane-1-carboxylic acid (ACC), Abscisic acid (ABA), and Salicylic acid (SA) concentrations were measured as described in the Section “Materials and Methods.” Ethylene evolution was measured from detached inflorescences as described in the Section “Materials and Methods.” The error bars represent the standard error of two biological replicates. ^∗^Indicates statistically significant (*p* < 0.006) between the L*er*-0 and *ag-1* inflorescences using Student’s *t*-test. FW, Fresh Weight. Student’s *t*-test at *p <* 0.05 showed that ethylene evolution between L*er*-0 and *ag-1* inflorescences was not significant.

To determine whether changes in plant hormones could be responsible for the delayed sepal senescence phenotype of *ag-1* plants, we measured JA, ACC (a precursor of ethylene), ABA, and SA concentrations in L*er*-0 and *ag-1* inflorescences (**Figures 3A,B**). In contrast with the wild type, JA concentration was significantly lower in *ag-1* inflorescences (**Figure 3A**). Concentrations of ACC, ABA and SA in *ag-1* inflorescences were not different from those in the wild type. Furthermore, the amount of ethylene evolved into the headspace from detached *ag-1* inflorescences was not significantly different from that of wild type inflorescences (**Figure 3C**). This was consistent with lowered JA concentration in the inflorescences causing the delayed senescence and abscission of the *ag-1* sepals.

### Methyl Jasmonate Treatment of *ag-1* Flowers Induces Sepal Yellowing and Abscission

**FIGURE 4:**
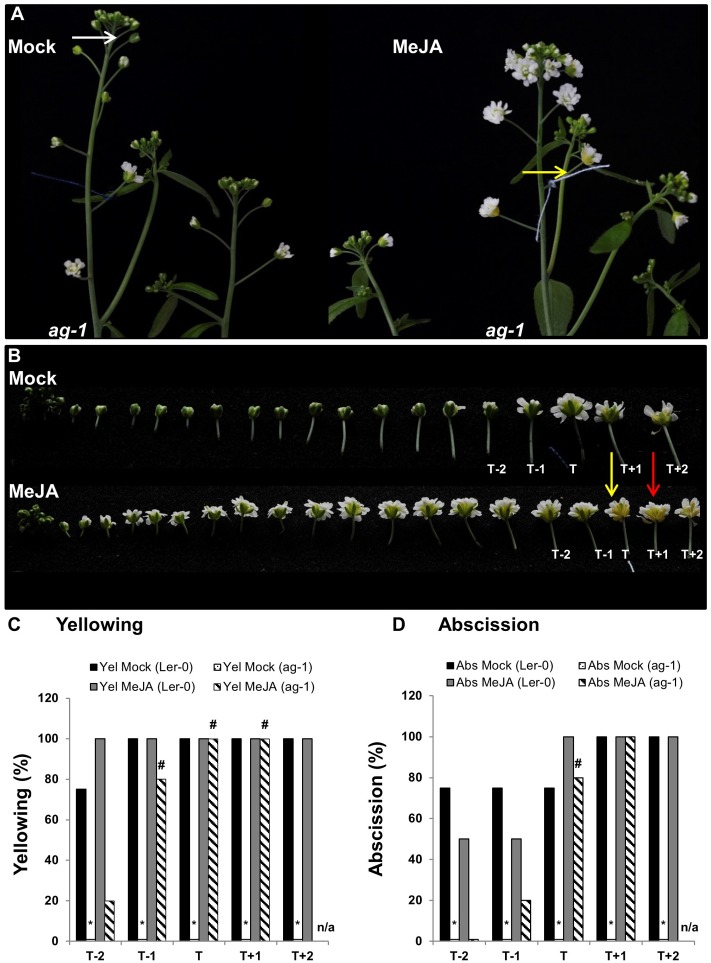
Exogenous application of MeJA to *ag-1* flowers. **(A)** Mock- and 100 μM MeJA-treated *ag-1* plants. Plants were grown under long-day conditions for 6 weeks and sprayed once a day with mock or MeJA solutions for 3 days. Flower buds with visible white petals were tagged with a cotton thread at day 0 of the MeJA or mock treatments and inflorescences were photographed after 3 days of treatment. **(B)** Flowers of mock- and 100 μM MeJA-treated *ag-1* inflorescences were detached and photographed. **(C)** Percentage sepal yellowing of Mock- and MeJA-treated *ag-1* and L*er*-0 flowers. **(D)** Percentage sepal abscission of Mock- and MeJA-treated *ag-1* and L*er*-0 flowers. White arrow indicates floral bud at stage 13 (Alvarez-Buylla et al., 2010), yellow arrows indicate flowers with senesced sepals, and red arrow indicates a flower with abscised sepals. T represents the tagged flower, T^-1^ and T^-2^ represent flowers younger than T, T^+1^ and T^+2^ represent flowers older than T. 0% datapoints are placed slightly above the X-axis. Statistical differences between mock treated wild type and mutants (indicated by ^∗^) and mock- and MeJA-treated mutant plants (indicated by ^#^) were calculated according to the Wilson score confidence limits for a percentage as calculated in Agresti and Coull, 1998 (Supplementary Table S2). The photographs shown are representatives of three (**C,D**; MeJA treated T^+1^ and T^+2^) or four to six biological replicates. Sen, Senescence; Abs, Abscission, n/a, not applicable.

To further investigate whether lack of JA was the cause of the *ag-1* delayed sepal yellowing and abscission we tested whether exogenously applied MeJA would accelerate sepal yellowing *in planta*. To do this, we used 6-week-old plants because of the ability to clearly stage flowers of *ag-1* and wild type. To describe the developmental stages of flowers in the following experiments we made use of the system described by Alvarez-Buylla et al. (2010). Six-week-old *ag-1* and L*er*-0 flowers of developmental stage 13 were tagged with cotton threads. At this stage white petals were visible among the sepals (Alvarez-Buylla et al., 2010). Plants were then sprayed with mock or MeJA solutions each day for 3 days. Three days post-treatment, sepal senescence and abscission phenotypes of the tagged flower and the four flowers surrounding the tagged flower were compared between the mock- and MeJA-treated plants of each genotype. The cotton-thread tagged flowers were designated as ‘T’ (for tagged) flowers, while the flanking two younger flowers were called T^-1^ and T^-2^ and the flanking two older flowers T^+1^ and T^+2^ (**Figure 4**).

The data showed that 100% of the T flowers on the MeJA-treated *ag-1* plants showed sepal yellowing compared to 0% for the T flowers of the mock-treated *ag-1* plants (**Figure 4C**). This difference is significant according to the Wilson score confidence limits for a percentage as calculated in Agresti and Coull (1998) (Supplementary Table S2). In addition, 20% of T^-1^ and 80% of the T flowers of MeJA-treated *ag-1* plants also displayed sepal abscission (**Figure 4D** and Supplementary Figure S1). All T^+1^ flowers of the MeJA-treated *ag-1* plants showed yellowing and abscission (**Figures 4C,D**). Eighty and twenty percent of the T^-1^ and T^-2^ flowers of the MeJA-treated *ag-1* plants showed sepal yellowing, respectively. In comparison, none of the equivalent flowers of the mock-treated *ag-1* plants had yellow or abscised sepals.

In contrast to the *ag-1* flowers, no significant differences were observed in the sepal yellowing and abscission phenotypes of the T^-2^ to T^+2^ flowers of the mock- and MeJA-treated L*er*-0 plants (**Figures 4C,D** and Supplementary Figure S2).

The data shows that exogenously applied MeJA complemented the delayed sepal yellowing and abscission phenotypes in the *ag-1* plants, further supporting the suggestion that decreased JA concentrations in *ag-1* sepals are responsible for the delayed sepal yellowing and abscission.

### JA Deficient Mutants Show Delayed Sepal Yellowing and Abscission

**FIGURE 5:**
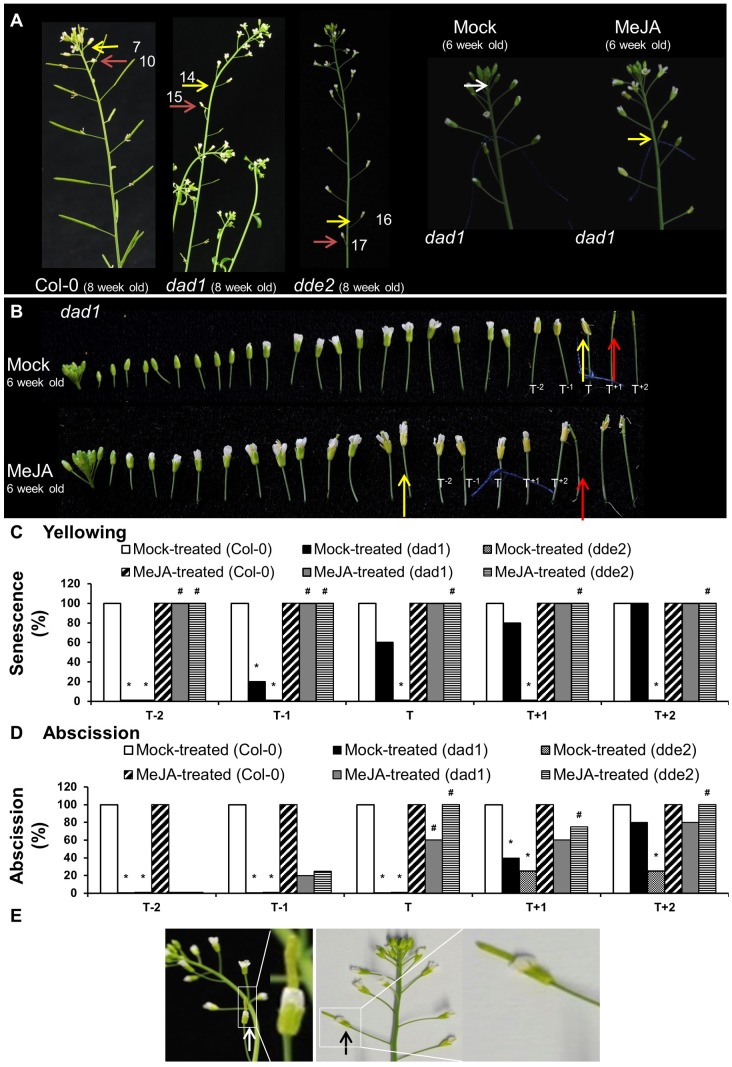
Exogenous application of MeJA to Col-0, *dad1* and *dde2* flowers. **(A)** Col-0, *dad1* and *dde2* plants were grown for 8 weeks and photographed. Mock and MeJA treated *dad1* plants were grown for 6 weeks and flower buds with visible white petals were tagged with a cotton thread at day 0 of MeJA or mock treatment. Plants were sprayed once a day with a mock solution or MeJA for 3 days. Inflorescences were photographed after 3 days of treatment. **(B)** Detached flowers of Mock- and MeJA-treated *dad1* inflorescences. **(C)** Percentage sepal yellowing of Mock- and MeJA-treated Col-0, *dad1* and *dde2* flowers. **(D)** Percentage sepal abscission of Mock- and MeJA-treated Col-0, *dad1* and *dde2* flowers. **(E)** *dad1* inflorescences showing hand-pollinated flowers developing into siliques (white and black arrows). The white arrow in **(A)** indicate a floral bud at stage 13 according to Alvarez-Buylla et al. (2010), yellow arrows indicate flowers with senesced sepals, red arrows indicate flowers with abscised sepals. T represents the tagged flower, T^-1^ and T^-2^ represent flowers younger than T, T^+1^ and T^+2^ represent flowers older than T. The numbers in **(A)** represent the flowering position according to the numbering system of Bleecker and Patterson, 1997. The photographs shown are representatives of four to six biological replicates. Sen, Senescence; Abs, Abscission, 0% datapoints are placed slightly above the *X*-axis. Statistical differences between mock treated wild type and mutants (indicated by ^∗^) and mock- and MeJA-treated mutant plants (indicated by ^#^) were calculated according to the Wilson score confidence limits for a percentage as calculated in Agresti and Coull (1998) (Supplementary Table S2).

To provide further evidence that the lack of JA was likely responsible for the delayed yellowing and abscission phenotypes of *ag-1* flowers, we examined the timing of sepal yellowing in the JA-deficient mutants *dad1* and *dde2*. DAD1/CHLOROPLASTIC LIPASE A1 produces linolenic acid, which is used as a substrate for JA production. DDE2/ALLENE OXIDE SYNTHASE controls the conversion of 13-HPOT, the first dedicated step of JA biosynthesis pathway, to unstable allene oxide (Creelman and Mullet, 1997; Mueller, 1997; Schaller and Weiler, 1997; Schaller et al., 1998). Sepals of flowers from 8-week-old wild type Col-0 plants yellowed at positions 7–8 and abscised at positions 9–11 whereas *dad1* sepals senesced at positions 13–16, and abscised at positions 15–17 (**Figure 5A**). Furthermore, the sepals of *dde2* were always more delayed in senescence than *dad1* and appeared to senesce just prior to abscission at positions 15–17 (**Figures 5A,C**). The delayed sepal senescence and abscission phenotypes of these JA deficient mutants suggest that the observed mutant phenotypes were caused by lack of JA. To test this further, we sprayed 6 week-old Col-0, *dad1* and *dde2* plants with mock and MeJA solutions. Before treatment, flowers of developmental stage 13 were tagged and after treatment sepal senescence and abscission phenotypes were noted for the tagged and adjacent flowers as described in the previous section (**Figures 5B–D** and Supplementary Figures S3–S5). All flowers of MeJA-treated *dad1* and *dde2* plants present at the positions T^-2^ to T^+2^ showed sepal yellowing (**Figure 5C**). Sepals of MeJA-treated T^-2^ and T^-1^ *dad1* and *dde2* flowers showed no or 20% abscission while 60% or more abscission was found in T to T^+2^ flowers (**Figures 5B,D** and Supplementary Figures S4, S5). In contrast to MeJA-treated flowers, mock-treated T^-2^ *dad1* and *dde2* flowers did not show sepal yellowing and abscission. Of the mock-treated *dad1* flowers, 20% of T^-1^ flowers showed sepal yellowing and this increased to 100% in T^+2^ flowers. Sepal abscission was first found in T^+1^ mock-tretated *dad1* flowers. Unexpectedly, we did not observe any yellowing in mock-treated *dde2* flowers of 6-week-old plants, even though abscission started from T^+1^ flowers (**Figures 5C,D** and Supplementary Figures S4, S5).

Regardless of the treatment, all the sepals of the T^-2^ to T^+2^ flowers of Col-0 plants were abscised (**Figure 5D** and Supplementary Figure S3). Closer inspection of T^-10^ to T^-3^ Col-0 flowers suggested that sepal yellowing and abscission was not enhanced upon the MeJA treatment (Supplementary Figure S3). This data suggests that MeJA treatment enhances sepal senescence and abscission in both *dad1* and *dde2* mutants, but not in Col-0 plants. The results from these JA defective mutants is consistent with the lack of JA being the cause of delayed sepal senescence and abscission in the *ag-1* mutant.

### Arabidopsis Sepal Yellowing and Abscission Does Not Dependent on Pollination

The MeJA treatment, in addition to complementing the delayed sepal yellowing and abscission phenotypes of the JA-deficient *dad1* mutant, also corrected defects in anther dehiscence, enabling pollination and silique development (Supplementary Figure S4B). Because silique development is closely associated with sepal yellowing and petal shatter, we investigated the role of reproductive development in accelerating sepal yellowing. For this purpose, we pollinated *dad1* flowers with the pollen from Col-0 flowers. Hand pollination of these mutants resulted in silique development and seed set, however, their sepals remained green and attached (**Figure 5E**), although ultimately they did yellow and abscise. This shows that sepal yellowing and abscission in these Arabidopsis flowers was not dependent on any pollination-mediated effects and suggests that that delayed sepal yellowing and abscission of *ag-1* is not due to lack of pollination.

## Discussion

Our study has highlighted a novel role for AGAMOUS in controlling senescence of the flower it helps specify. Researchers have for many years demonstrated through physiological studies that flower senescence is regulated by plant hormones such as ethylene, ABA and cytokinins, and by source-sink relationships (Rogers, 2013). However, the key genetic regulators that are critical for initiating age-dependent flower senescence have remained elusive. Here we suggest that for the ephemeral flower of Arabidopsis, the MADS box transcription factor, AG, plays a role in the regulation of sepal senescence by inducing JA production in the stamens.

### MADS Box Genes Regulate Flower Longevity in Arabidopsis

Control of flower longevity by MADS box transcription factors has been found by multiple studies. Ectopically expressed *AGL15*, *AGL18*, and *AGL42* delayed sepal yellowing and perianth abscission in Arabidopsis (Fernandez et al., 2000; Adamczyk et al., 2007; Chen et al., 2011; Deng et al., 2011). Chen et al. (2011) found that ectopically expressed AGL42 reduced transcript abundance of the repressor-encoding genes *ETHYLENE RESPONSE DNA-BINDING FACTOR 1* (*EDF1*) and *EDF2*. The authors subsequently revealed that ectopic AGL42 expression delayed floral senescence by increasing transcript abundance of an *ETHYLENE RESPONSE FACTOR (AtERF21*), whose product activated a mechanism that suppressed transcript induction of the *EDF* genes (Chen et al., 2015). This led them to propose that flower senescence in Arabidopsis is initiated during flower aging because of *AGL42* transcript abundance declining. This results in lowered transcript accumulation of *AtERF21/FUF1*, preventing its product from activating a suppressor of *EDF* transcription. The increased EDF transcription then results in lowered transcript abundance of a suppressor of senescence. In addition, the authors found that perianth senescence and abscission was further delayed when the *EDFs* ectopic expression occurred in the *ethylene response 1* (*etr1*) and *ethylene-insensitive 2* (*ein2*) backgrounds. This suggests that the programs are under the regulation of an additional component other than ethylene.

Fernandez et al. (2000) proposed an ethylene-independent role of *AGL15* in regulating Arabidopsis perianth senescence and abscission. The extent in the delay in flower organ abscission in the JA receptor (*dab4*) and biosynthesis mutant (*aos*) was greater than the delay observed in *etr1-1* or *ein2-1* mutants, suggesting that JA plays a major role in perianth abscission (reviewed by Kim, 2014). Kim et al. (2013) further found an increased transcript abundance of *AGL15* in the JA non-responsive *coi1* mutant, which exhibits delayed floral organ abscission. A recent study by Patharkar and Walker (2015) showed that *AGL15* overexpression resulted in altered expression of genes involved in abscission. Notably, this included the reduced expression of receptor-like protein kinase *HAESA* that has been shown to play a role in floral organ abscission. At the time of flower opening a transient increase in *AGL15* transcripts was found to be enough for delaying flower senescence and abscission (Fang and Fernandez, 2002). Kim et al. (2013) also speculated that delayed senescence and abscission in *AGL15* overexpression mutants was due to altered levels of senescence regulating hormones such as cytokinin and auxin.

Thus, a reduced JA presence and response correlates with increased AGL15 transcript abundance, while increased AGL15 levels correlate with delayed perianth senescence and abscission. Therefore, it is conceivable that AGAMOUS may regulate sepal senescence and abscission by controlling JA production and AGL15 expression. Lower JA quantities in *ag-1* flowers may result in increased *AGL15* transcript and subsequently delayed flower development. Interestingly *AGL15* transcript abundance was found to be increased after 7 days of AG activation in AG-GR transgenic plants where AG expression was under the external control of glucocorticoids (Gomez-Mena et al., 2005). Fang and Fernandez (2002) demonstrated that siliques of plants overexpressing AGL15 remained green compared to the wild-types. The AG-mediated increased AGL15 activity may have a role in ensuring proper seed maturation in developing siliques so that siliques do not dehisce until the fruit has matured. Thus we suggest that AG regulates both positive and negative regulators of flower maturity to ensure a timely flower development and death.

### Role of JA in Sepal Senescence and Abscission

Many studies have demonstrated the critical roles of ethylene and ABA in regulating senescence and abscission of leaves and flowers (Abeles and Rubinstein, 1964; Jackson and Osborne, 1970; Brown, 1997; Rungruchkanont, 2011). By contrast, the evidence for JA regulating age-related senescence processes has been ambiguous (Jibran et al., 2013). On the one hand, JA concentrations increase in senescing Arabidopsis leaves (Seltmann et al., 2010), exogenously applied JA accelerates senescence of attached leaves (He et al., 2002), JA treatment increases expression of chlorophyll catabolic genes (Reinbothe et al., 2009) and JA represses RUBISCO ACTIVASE, an enzyme involved in carbon fixation, in a COI1-dependent manner (Shan et al., 2011). However, on the other hand, JA-deficient mutants such as *opr3* and *dde2*/*aos* do not show delayed leaf senescence (Stintzi and Browse, 2000; Park et al., 2002). Thus the role of JA as a regulator of developmental senescence has remained questionable (Jibran et al., 2013). Kim et al. (2013) have, however, recently demonstrated a role for JA in petal abscission and here we provide further evidence in support of a role for JA during senescence, by using Arabidopsis sepals as a model.

The sepals of *dde2* flowers showed a greater delay in senescence compared to *dad1* sepals (**Figure 5A**). This could be because DAD1 specifically contributes to JA production in the flower and does not affect wound-induced and basal JA production (Ishiguro et al., 2001; Hyun et al., 2008), whereas *dde2* plants are completely devoid of JA (Stintzi and Browse, 2000). Thus it is possible that in *dad1* mutants JA translocation from other plant parts may have caused the earlier sepal senescence and abscission. The function of JA signaling components such as COI1, JAZ proteins, and JA-Ile in the regulation of flower senescence is still unclear and research in this area deserves attention.

MeJA treatment opened many flowers along the stalk rapidly, but only the older unopened florets at time of treatment yellowed. This strongly suggests presence of an age-related component to senescence of the flower, which involves the flowers developing an increased competence to senesce in response to JA. By contrast, the oldermost florets of the mock treated *ag-1* flowers were not yellow after the 3 days. This suggests that these florets had developed a competence to senesce in response to JA, but had not done so because of the lack of JA production in their flowers. This clearly reveals an age-related increase in competence to respond to this hormone.

### Arabidopsis Sepal Senescence Does Not Depend on Pollination or a Fertilization-Associated Ethylene Burst

Pollination and fertilization in many plants, including petunia, daffodil, tobacco and carnation, cause a burst in ethylene production that induces the different flower organs to wilt and/or abscise (Hunter et al., 2004; Jones et al., 2007; van Doorn and Woltering, 2008). The rise in ethylene is caused mainly by the reproductive organs, particularly following pollination. By contrast, the sepals produce low amounts of the hormone and only after production by the reproductive organs has started. Ethylene produced by the reproductive organs may be what increases sepal sensitivity to ethylene and may induce sepal yellowing (Tian et al., 1994). Since *ag-1* flowers do not have reproductive organs, the absence of an ethylene burst may be the reason for the delayed sepal yellowing. Indeed, it has been found in various studies that the removal of reproductive organs can delay senescence, presumably due to the absence of sink tissue or decreased ethylene production (Hensel et al., 1994; Tian et al., 1994; Nooden and Penney, 2001). Here we showed that fertilization and silique development occurred in hand-pollinated, jasmonate-deficient flowers. However, sepal senescence was still delayed, suggesting that pollination-based signals, that typically involve ethylene, do not affect longevity of the Arabidopsis flower or have only a minor effect. This suggestion is consistent with the small delay in abscission seen for the ethylene-insensitive *ein2-1* mutant of Arabidopsis (Kim et al., 2013) and contrasts with the larger delay in senescence and abscission observed in the jasmonate-deficient flowers of mutants *ag-1*, *dde2* and *dad1* (**Figures 4**, **5**; Kim et al., 2013).

Our results are further supported by Azumi et al. (2002) who identified a meiotic mutant called *solo dancer* (*sds*) that is both male and female sterile but otherwise normal in plant and flower development. The *sds* mutant flowers do not show delayed sepal senescence phenotypes (**Figures 1A–C**, Azumi et al., 2002). Similarly, Huang et al. (2016) showed in their **Figures 5A–C** that the sepals of the sterile SDS::SDS-BARNASE lines senesced and abscised at a similar time to wild type. Further, Wang et al. (2008) reported that the *mpk3+/-mpk6-/-* sterile mutants were somewhat delayed in senescence, but not as much as the *ag-1* mutant shown here. Moreover, *mpk6-/-* mutants have a delayed leaf senescence phenotype (Zhou et al., 2009) which may affect sepal senescence as well, although this was not studied. Therefore, from these studies we conclude that infertility in Arabidopsis is not tightly linked with delayed sepal and petal senescence and abscission in Arabidopsis.

### Functions for AGAMOUS in Flower Development

**FIGURE 6:**
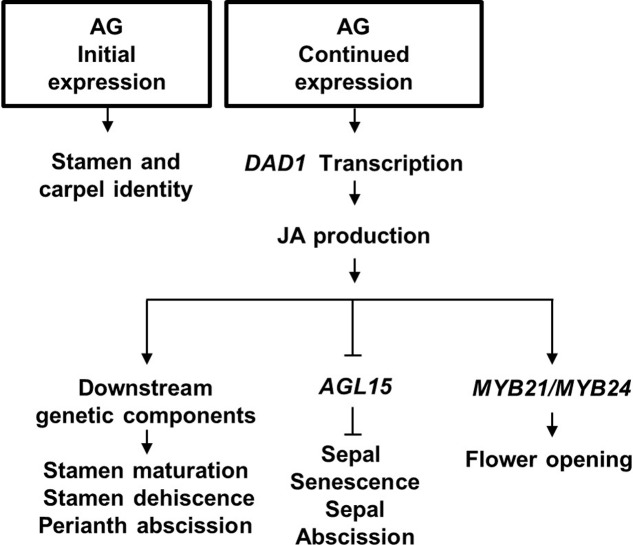
Proposed model for Arabidopsis flower development. Initial AGAMOUS (AG) expression is required for stamen and carpel identity of the innermost whorls of the flower, whereas its late expression causes Jasmonic acid (JA) synthesis in the mature stamens by directly binding to the JA production *DAD1* gene. JA in the flower ensures stamen maturation, and stamen dehiscence. JA is proposed to derepress sepal senescence and abscission by inhibiting *AGL15* expression.

In conclusion, we propose that AG has a central role in initiation, development and senescence of the flower it helps specify. The model in **Figure 6** describes that (1) AG activity initially specifies stamen and carpel identity in the floral primordia (reviewed in Wellmer et al., 2014); (2) its continued expression drives late-stage stamen development by binding to the *DAD1* promoter to induce jasmonate biosynthesis (Ito et al., 2007); (3) jasmonate then drives petal and sepal expansion possibly by regulating *AGL15* expression levels and water transport, resulting in flower opening, stamen maturation and dehiscence (Ishiguro et al., 2001). We further propose that JA signaling initiates sepal yellowing and perianth abscission through suppressing transcript accumulation of *AGL15* (Kim et al., 2013; our data), independently of its effects on pollination and fertilization (**Figure 6**).

This model predicts that the timing of AG expression is important for the regulation of sepal senescence and conditional AG expression may help establish temporal expression effects. Ectopic overexpression of AG in a 35S:AG-GR line caused sepals to develop carpelloid features (Sun et al., 2009) and therefore this approach may not yield answers. An amiRNAi knock-down approach may be more suitable and Ó’Maoiléidigh et al. (2013) showed that inducible amiRNAi flowers had sepals that were more green than the control flowers. Another avenue for future research would be to establish the roles of B class genes such as *APETALA3* and *PISTILLATA* in sepal senescence. The mutant *unusual floral organs* (*ufo*) is deficient in APETALA3 and PISTILATA and has flowers in which the second and the third whorls are composed of a mosaic combination of sepals, petals, stamens and carpels. Nevertheless, sepals of the first whorl appear normal (Levin and Meyerowitz, 1995). The floral phenotype of the *ufo* mutant suggests that *ufo* sepals were not delayed in degreening, although the inflorescence phenotype is altered (Levin and Meyerowitz, 1995) and therefore not that easy to use for sepal senescence studies. Regardless, this study is consistent with our hypothesis that flowers with functional stamens will senesce in a timely manner.

## Author Contributions

RJ, DH, and PD jointly conceived the study, acquired and interpreted the data, wrote the manuscript and assisted with manuscript revision. JC and JT acquired and analyzed data and commented on the manuscript. All the authors have read and approved the final version of the manuscript.

## Conflict of Interest Statement

The authors declare that the research was conducted in the absence of any commercial or financial relationships that could be construed as a potential conflict of interest.

## Footnotes

1 http://www.arabidopsis.org
