# Supplementary material for: Arabidopsis AGAMOUS Regulates Sepal Senescence by Driving Jasmonate Production
Source: Front Plant Sci. 2017 Dec 11;8:2101. doi: 10.3389/fpls.2017.02101 (PMC5732193; doi:10.3389/fpls.2017.02101)
Supplement: Supplementary file 1 [file Image_1.pdf]

Supplementary Material

Arabidopsis AGAMOUS regulates sepal senescence by driving jasmonate production

Rubina Jibran, Jibran Tahir, Janine Cooney, Donald A. Hunter, Paul P. Dijkwel

Supplementary Table S1: Multiple Reaction Monitoring (MRM) transitions used for plant hormones and their isotopically labelled internal standard analogues

Q1	Q3	Compound	DP	EP	CE	CXP
137.1	92.8	salicylic acid	-140	-10	-22	-9
209.0	59.0	jasmonic acid	-25	-10	-25	-15
263.0	153.0	abscisic acid	-25	-10	-17	-15
141.1	97.0	[$^2H_4$]salicylic acid	-35	-10	-30	-15
214.0	62.0	[$^2H_5$]jasmonic acid	-25	-10	-25	-15
269.0	159.0	[$^2H_6$]abscisic acid	-25	-10	-17	-15
102.1	56.2	1-aminocyclopropane-1-carboxylic acid	30	10	15	15

DP = declustering potential, EP = entrance potential, CE = collision energy, CXP = collision cell exit potential

Supplementary Table S2: 95% Wilson score confidence limits for a percentage as calculated in Agresti and Coull (1998). The Wilson score confidence limits for a percentage was calculated to estimate population proportion showing senescence and abscission phenotypes in mock-treated L*er*-0, *ag-1*, Col-0, *dde2* and *dad1* plants and MeJA-treated L*er*-0, *ag-1*, Col-0, *dde2* and *dad1* plant. The confidence interval was calculated for Fig.4 C, D, Fig. 5C, D, E, supplementary Fig.S2, S3, S4, S5 and S6 by using 4 or 6 biological replicates.

Number of plants showing phenotype	Total number of replicates	Percentage	Lower limit	Upper limit
0	4	0	0	48.99
1	4	25	4.559	69.94
2	4	50	15	85
3	4	75	30.06	95.44
4	4	100	51.01	100
0	6	0	0	39.03
1	6	16	3	56.35
2	6	33	9.67	70.00
3	6	50	18.7	81.23
4	6	66	29.99	90.32
5	6	83	43.64	96.99
6	6	100	60.96	100

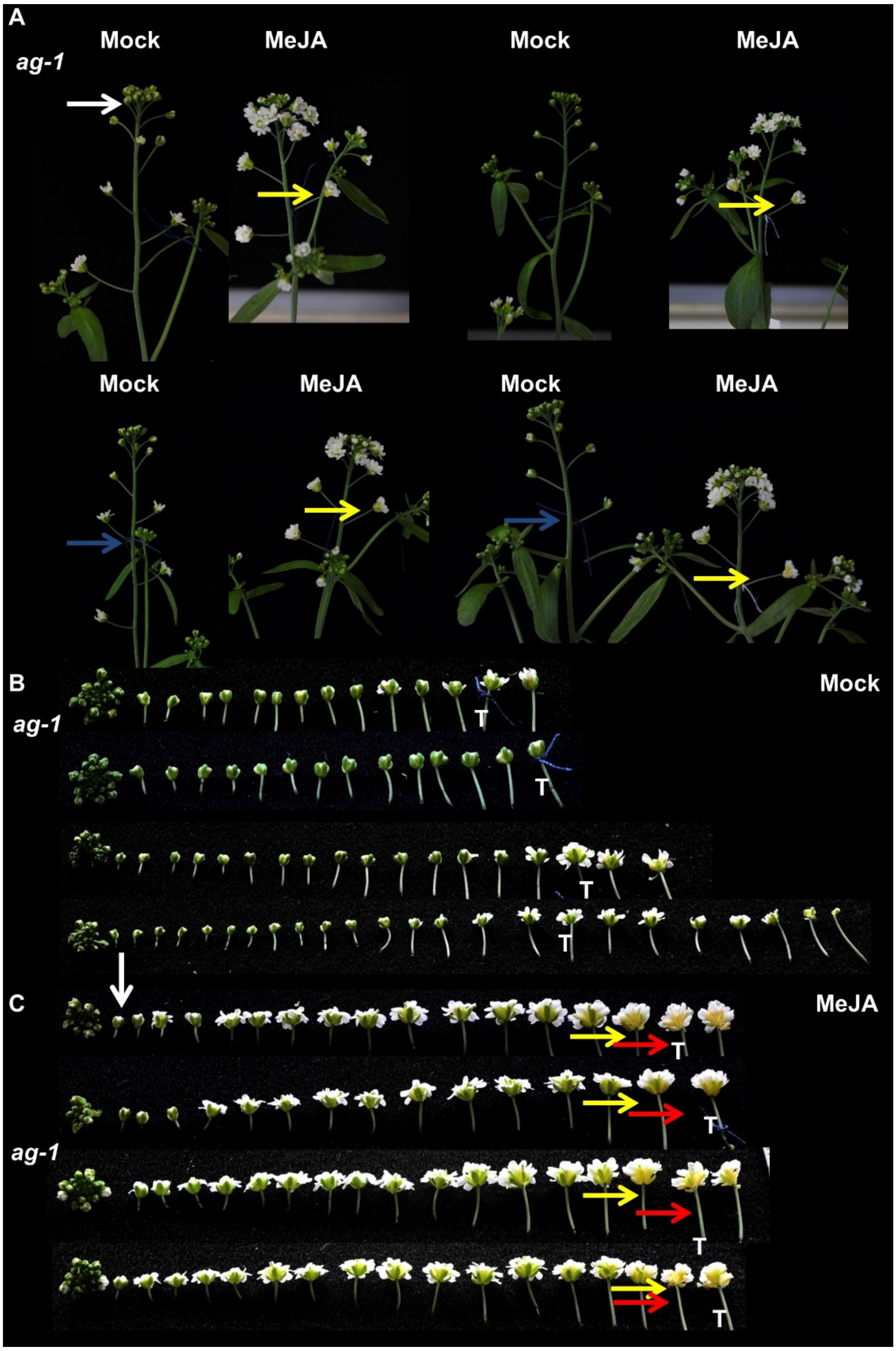

Supplementary Figure S1. Exogenous application of MeJA to *ag-1* flowers. (**A**) Mock- and 100 µM MeJA-treated *ag-1* flowers. Flower buds with visible white petals (stage 13, Alvarez-Buylla *et al.*, 2010) were tagged with a cotton thread at day 0 of the MeJA or mock treatments and inflorescences were photographed after three days of treatment. (**B**, **C**) Flowers of mock- and 100 µM MeJA-treated *ag-1* inflorescences were detached and photographed. Plants were grown under long day conditions for 6 weeks and sprayed once a day with mock or MeJA solution for three days. White arrows indicate floral buds at stage 13, yellow arrows indicate flowers with senesced sepals, and red arrows indicate flowers with abscised sepals. T represents the tagged flower.

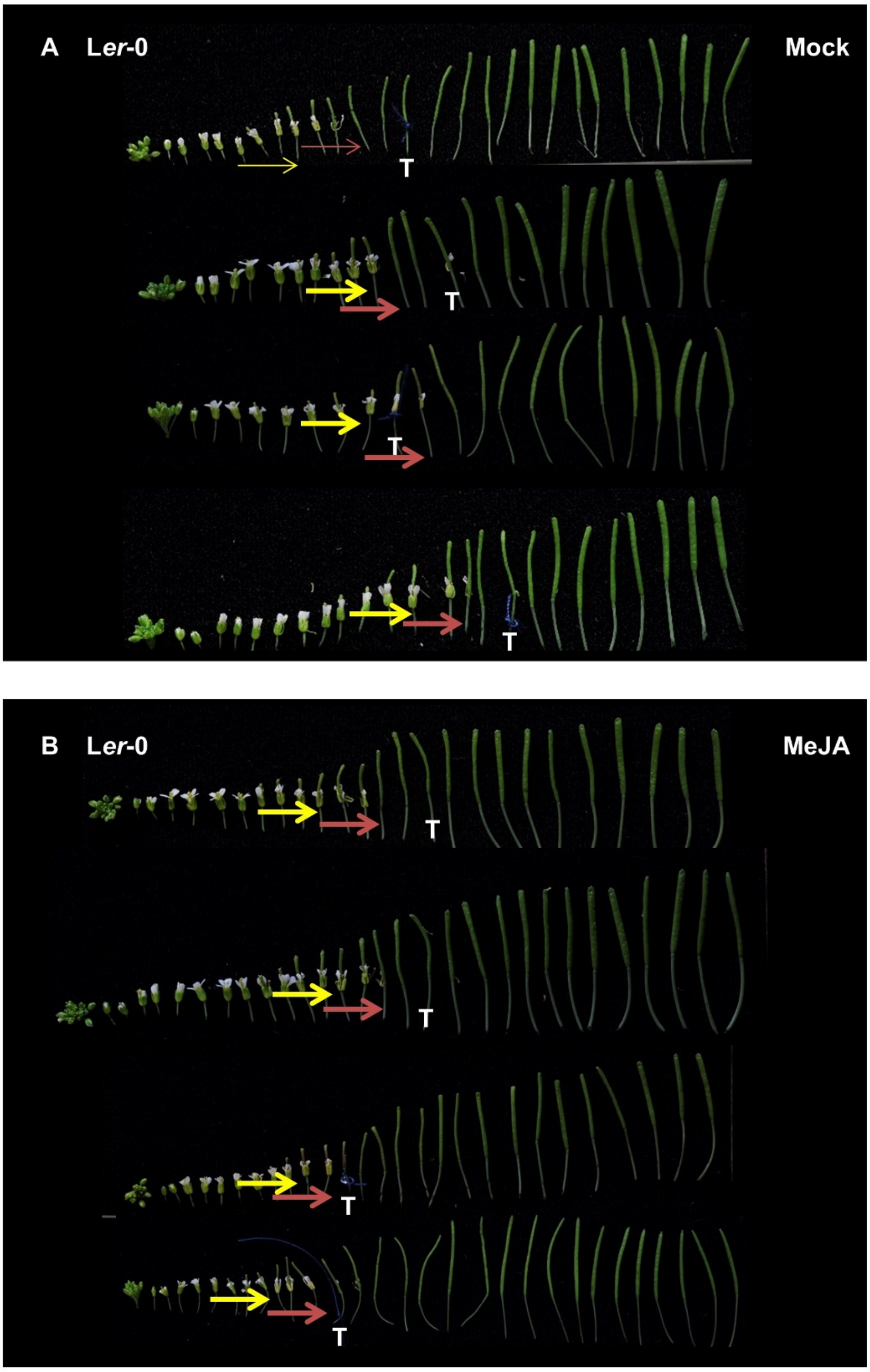

Supplementary Figure S2. Exogenous application of MeJA to L*er*-0 flowers. (**A**, **B**) Mock- and 100 µM MeJA-treated L*er*-0 flowers and siliques. Flower buds with visible white petals were tagged (stage 13, Alvarez-Buylla *et al.*, 2010) with a cotton thread at day 0 of the MeJA or mock treatment and inflorescences were photographed after three days of treatment. Flowers of mock- and 100 µM MeJA-treated L*er*-0 inflorescences were detached and photographed. Plants were grown under long day conditions for 6 weeks and sprayed once a day with MeJA or a mock solution for three days. Yellow arrows indicate flowers with senesced sepals and red arrows indicate flowers with abscised sepals. T represents the tagged flower.

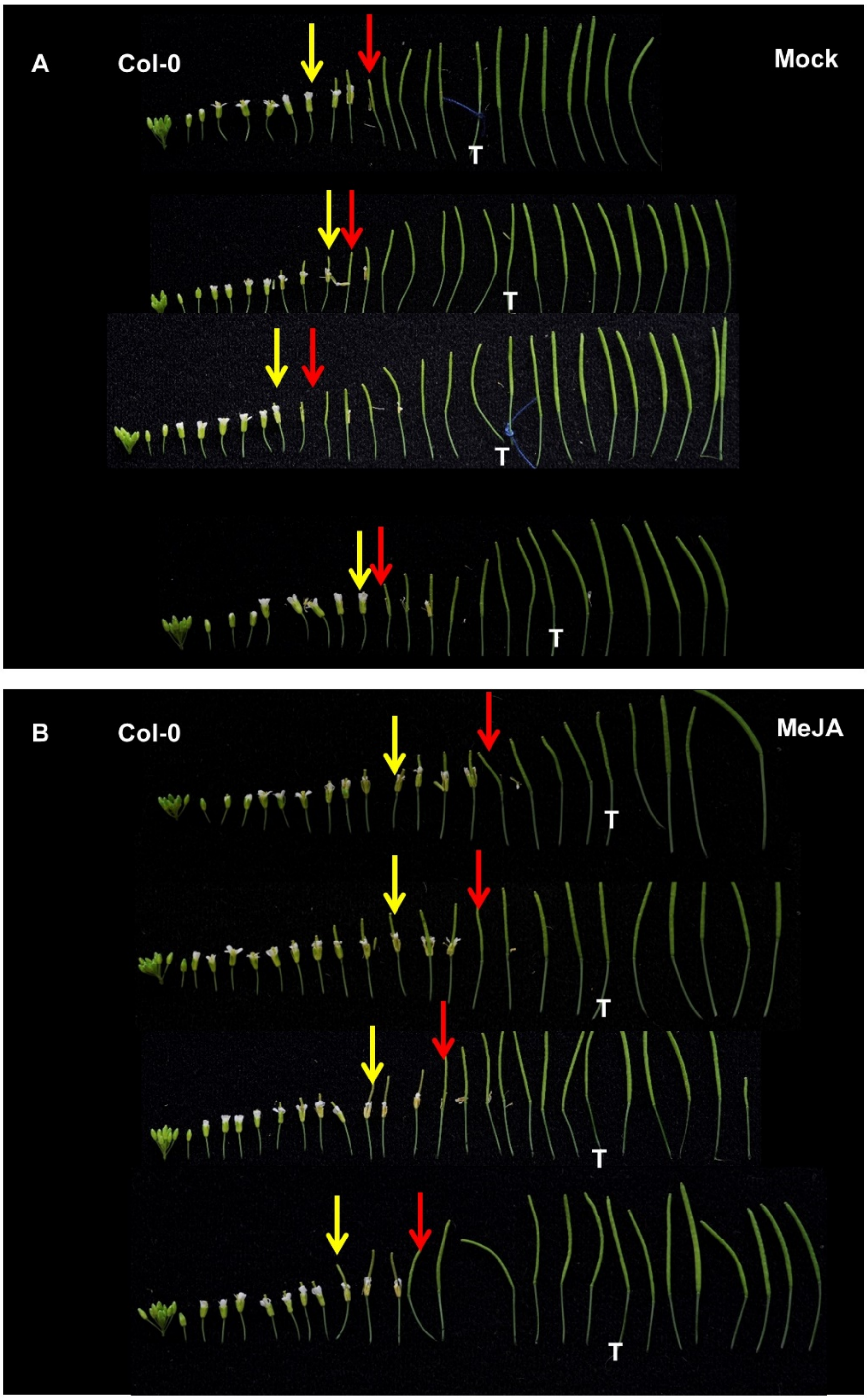

Supplementary Figure S3. Exogenous application of MeJA to Col-0 flowers. (**A**, **B**) Mock- and 100 µM MeJA-treated Col-0 flowers and siliques. Flower buds with visible white petals (stage 13, Alvarez-Buylla *et al.*, 2010) were tagged with a cotton thread at day 0 of the MeJA or mock treatments and inflorescences were photographed after three days of treatment. Flowers of mock- and 100 µM MeJA-treated Col-0 inflorescences were detached and photographed. Plants were grown under long day conditions for 6 weeks and sprayed once a day with MeJA or a mock solution for three days. Yellow arrows indicate flowers with senesced sepals, and red arrows indicate flowers with abscised sepals. T represents the tagged flower.

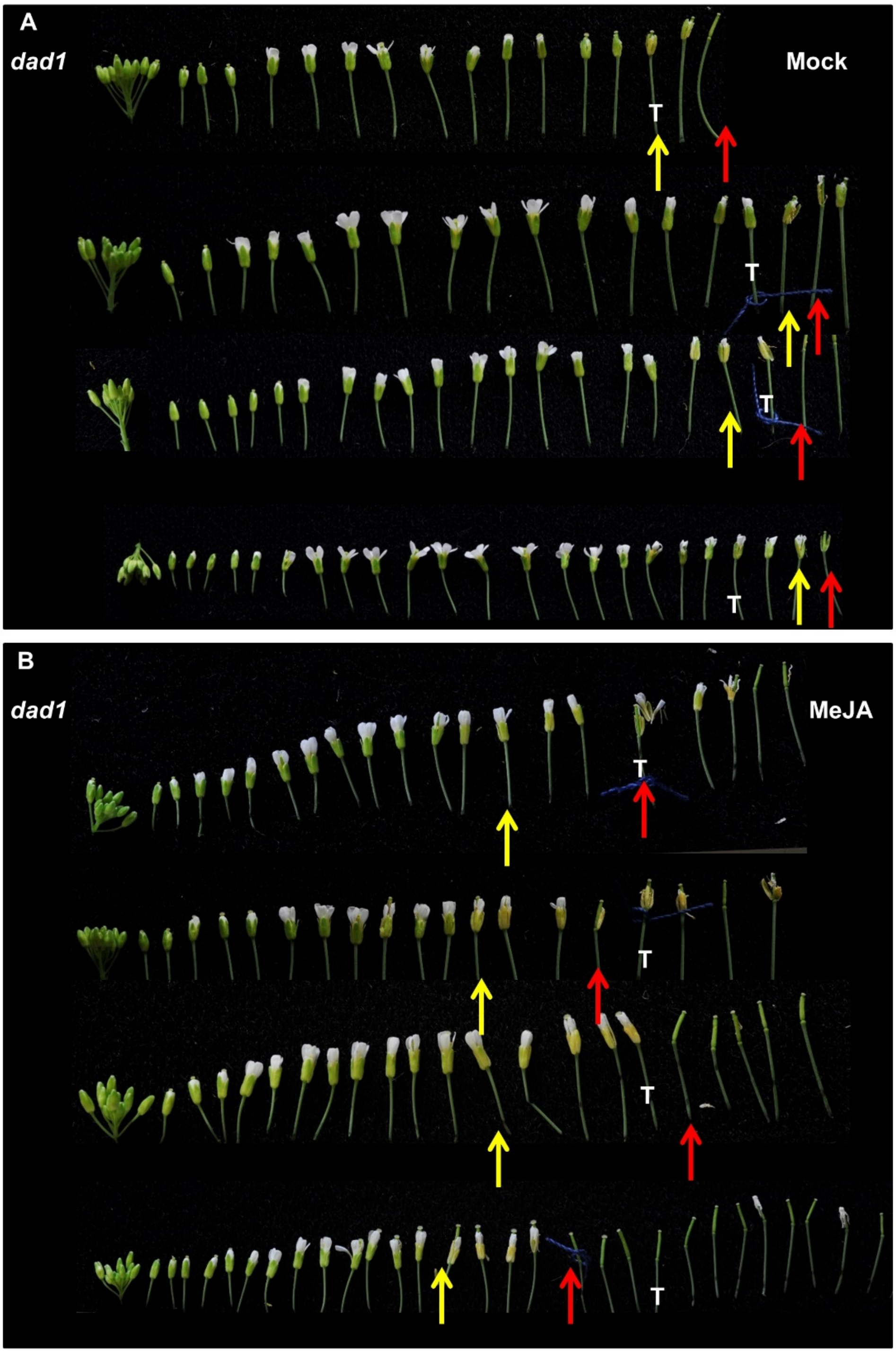

Supplementary Figure S4. Exogenous application of MeJA to *dad1* flowers. (**A**, **B**) Mock and 100 µM MeJA-treated *dad1* flowers. Flower buds with visible white petals were tagged (stage 13, Alvarez-Buylla *et al.*, 2010) with a cotton thread at day 0 of the MeJA or mock treatments and inflorescences were photographed after three days of treatment. Flowers of mock- and MeJA-treated *dad1* inflorescences were detached and photographed. Plants were grown under long day conditions for 6 weeks and sprayed once a day with MeJA or a mock solution for three days. Yellow arrows indicate flowers with senesced sepals, and red arrows indicate flowers with abscised sepals. T represents the tagged flower.

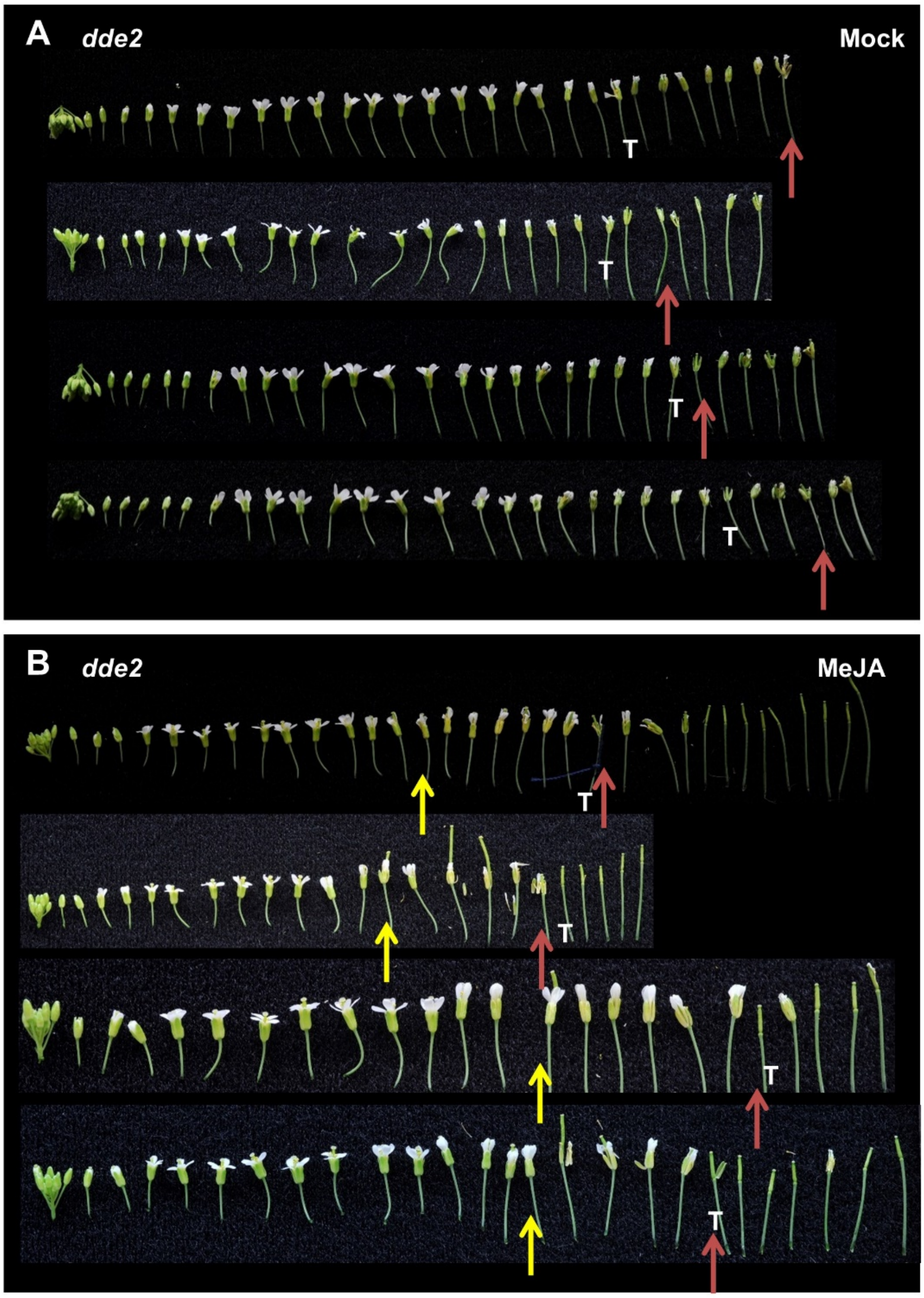

Supplementary Figure S5. Exogenous application of 100 µM MeJA to *dde2* flowers. (**A**, **B**) Mock and 100 µM MeJA-treated *dde2* flowers. Flower buds with visible white petals (stage 13, Alvarez-Buylla *et al.*, 2010) were tagged with a cotton thread at day 0 of the MeJA or mock treatments and inflorescences were photographed after three days of treatment. Flowers of mock- and MeJA-treated *dde2* inflorescences were detached and photographed. Plants were grown under long day conditions for 6 weeks and sprayed once a day with MeJA or a mock solution for three days. Yellow arrows indicate flowers with senesced sepals, and red arrows indicate flowers with abscised sepals. T represents the tagged flower.
